# A young patient with heart failure was diagnosed with extra-adrenal paraganglioma: a case report

**DOI:** 10.1186/s12872-022-03026-5

**Published:** 2022-12-29

**Authors:** Jing Zhang, Lihua Cao, Lina Yan, Cong Jin, Dan Zhang

**Affiliations:** Respiratory Department, The Second Hospital of Dalian Medical University, 467 Zhongshan Road, Shahekou District, Dalian, Liaoning China

**Keywords:** Paraganglioma, Extra-adrenal gland, Heart failure, Case report

## Abstract

**Background:**

We present a case of pelvic paraganglioma that presented with heart failure as the primary symptom.

**Case presentation:**

A 35-year-old man was admitted to hospital due to heart failure. Contrast-enhanced pelvic CT showed mass shadows in the posterior wall of the bladder and multiple enlarged lymph nodes in the retroperitoneal area. Ultrasound-guided puncture was performed, and the pathologic diagnosis was extra-adrenal paraganglioma. The patient refused any chemotherapy and died within six months of diagnosis.

**Conclusion:**

The possibility of neuroendocrine-related tumors, for example paragangliomas, should be considered in young patients with heart failure, especially those with concomitant hypertension and diabetes.

## Introduction

Paragangliomas are extra-adrenal non-epithelial neuroendocrine tumors originating along the sympathetic and parasympathetic nerve chains [1]. The WHO (2004) defines tumors originating from the adrenal medulla as pheochromocytoma, and tumors originating from the sympathetic nerve and parasympathetic nerve as paragangliomas [2]. Paragangliomas are rare and can occur in the sympathetic nerve chain of the abdomen, chest, neck, or bladder [3]. The reported incidence of paraganglioma is 2–8 per million per year [4]. Paragangliomas can be classified into functional and non-functional tumors. Functional tumor secrete catecholamines, and often cause symptoms such as hypertension, palpitations, and excessive perspiration. Nonfunctional paragangliomas are often detected due to symptoms associated with tumor compression. In this paper, we report a case of extra-adrenal paraganglioma presenting with heart failure in order to improve the understanding of this disease. This is a rare paraganglioma described with heart failure in a so young patient as the primary clinical presentation.

## Case presentation

A 35-year-old man was admitted to the hospital in May 2019 with chief complaints of fatigue and cough for 15 days, and dyspnea for 5 days. The patient had developed a bout of cold 15 days back followed by general malaise and fatigue, and had felt better after self-medication. 5 days ago, he developed dyspnea with no obvious cause, accompanied by chest tightness, and could not lie supine at night. Dyspnea was mainly in the supine position, and was relieved in sitting and standing position; in addition, there was significant reduction in activity tolerance. He had a history of hyperhidrosis for more than 10 years, which had aggravated in the last 4 years.

4 years ago, he was found to have hyperglycemia and high blood pressure, both of which were not well controlled. His father died because of bone cancer at the age of 30 (details are unknown). On physical examination, he was conscious and well oriented with a blood pressure of 147/102 mmHg, and heart rate of 122 beats per minute. No rales were heard in bilateral lungs. The heart rhythm was regular and no abnormal murmur was heard in the precordium. Mild edema was found in lower extremities.

**Fig. 1:**
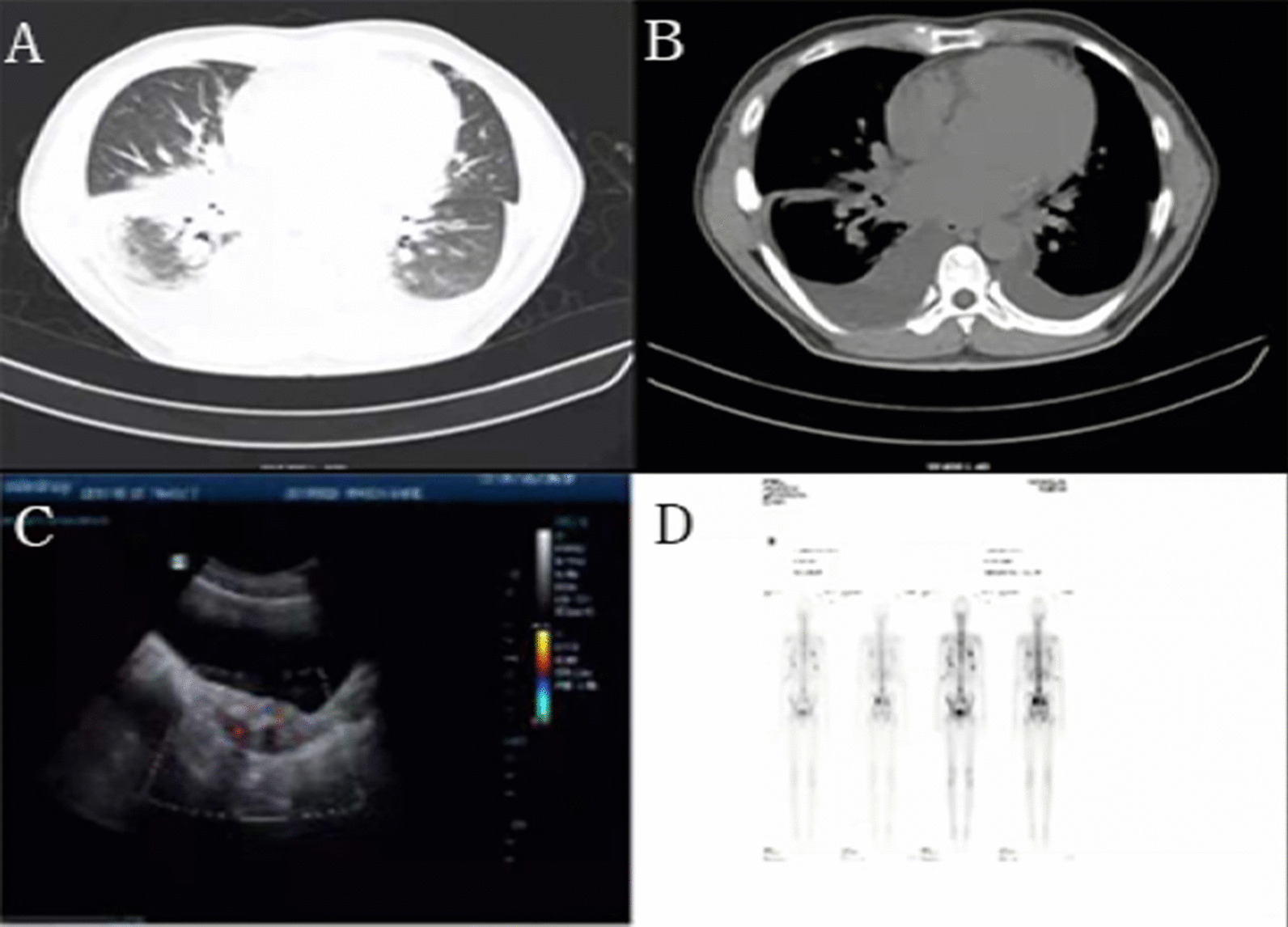
**A** and **B** Chest computed tomography showing bilateral pleural cavity and right interlobar fissure effusion. **C** Renal ureteral ultrasound showing pelvic hypoecho. **D** Bone ECT (Emission Computed Tomography) showing multiple bone metastases throughout the body

**Fig. 2:**
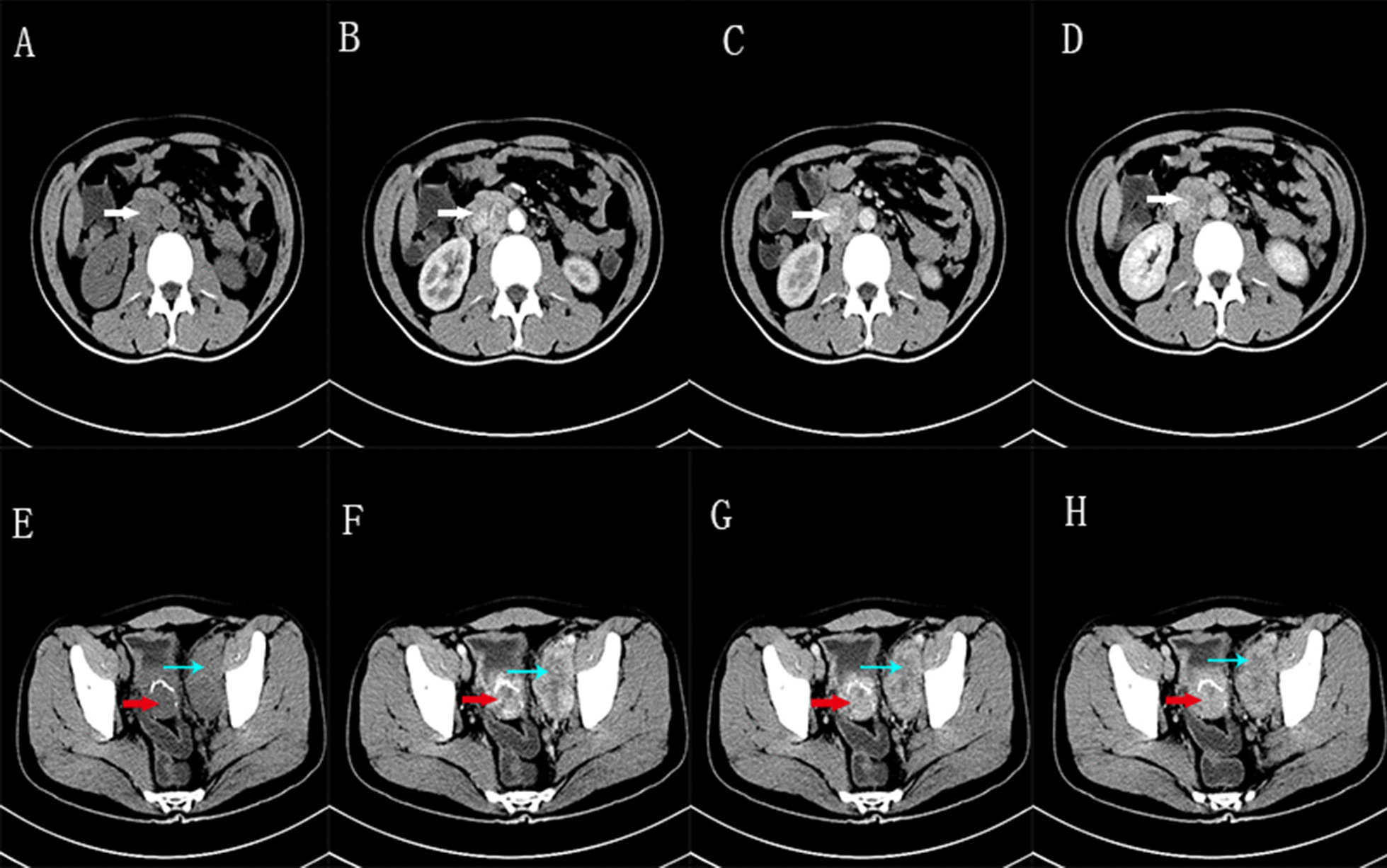
Upper, lower abdomen, pelvic cavity enhanced CT. **A** and **E** Plain scan; **B** and **F** arterial phase; **C** and **G** venous phase; **D** and **H** delayed phase. Blue and white arrows indicate enlarged lymph nodes. Red arrows indicate the mass shadow of the posterior wall of the bladder

At admission his BNP level was 350.50 pg/mL. Blood gas analysis showed type I respiratory failure. His 24-h urinary VMA level was 331.5 µmol/L (reference range, 0–60.6 µmol/L). Serum levels of aldosterone, cortisol, adrenocorticotropic hormone (16:00), metanephrine and norepinephrine were normal, while adrenocorticotropic hormone (8:00) was high slightly. (Table 1). Chest CT showed bilateral pleural effusion (Fig. 1A, B). Pathological examination of pleural effusion showed many atypical epithelioid cells, most of which were scattered while a few were arranged in clusters. Based on the characteristics of the cells, mesothelial hyperplasia or mesothelioma could not be excluded. CA125 level in pleural effusion was more than 600 U/mL and the CA125 level in blood was 401.03 U/mL (0–35 lU/mL). Renal ureteral ultrasound revealed a pelvic hypo-echoic mass (Fig. 1C). Enhanced CT of abdomen and pelvic cavity showed a mass in the posterior wall area of the bladder (size: 4.2 × 2.1 × 6.4 cm) consistent with a malignant tumor. Multiple enlarged retroperitoneal nodules (larger ones were approximately 59 × 34 mm in dimensions) were also detected, which were considered as metastasis (Fig. 2). Enhanced CT of adrenal gland showed no abnormality. ECT suggested multiple bone metastases throughout the body (Fig. 1D).

**Table 1:** Levels of catecholamine hormones and metabolites

Hormone	Value	Unit	Normal reference range
Adrenocorticotropic hormone	49.31	pg/ml	6–40
Adrenocorticotropic hormone 16:00	13.4	pg/ml	3–30
Metadepinephrine	0.21	nmol/l	0–0.3
noradrenaline	< 0.2	nmol/l	0–0.6
24-h urinary vanilmandelic acid	331.5	µmol/l	0–60.6

**Fig. 3:**
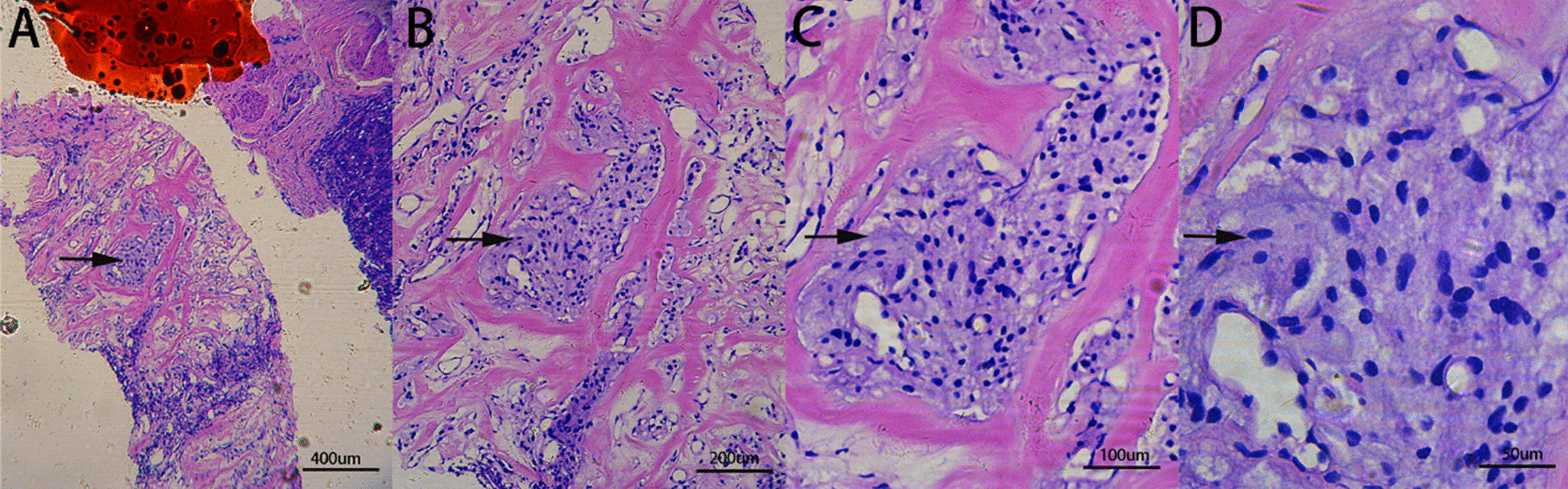
HE stained sections. Black arrows indicate the nest-like cell masses in the fibrous connective tissue. (**A** to **D** represents magnification ×50, ×100, ×200, ×400 respectively). Type of equipment: microscope: LEICA DM 2000 LED; cameras: LEICA MC 170 HD. Acquisition software: LASV4.10.

**Fig. 4:**
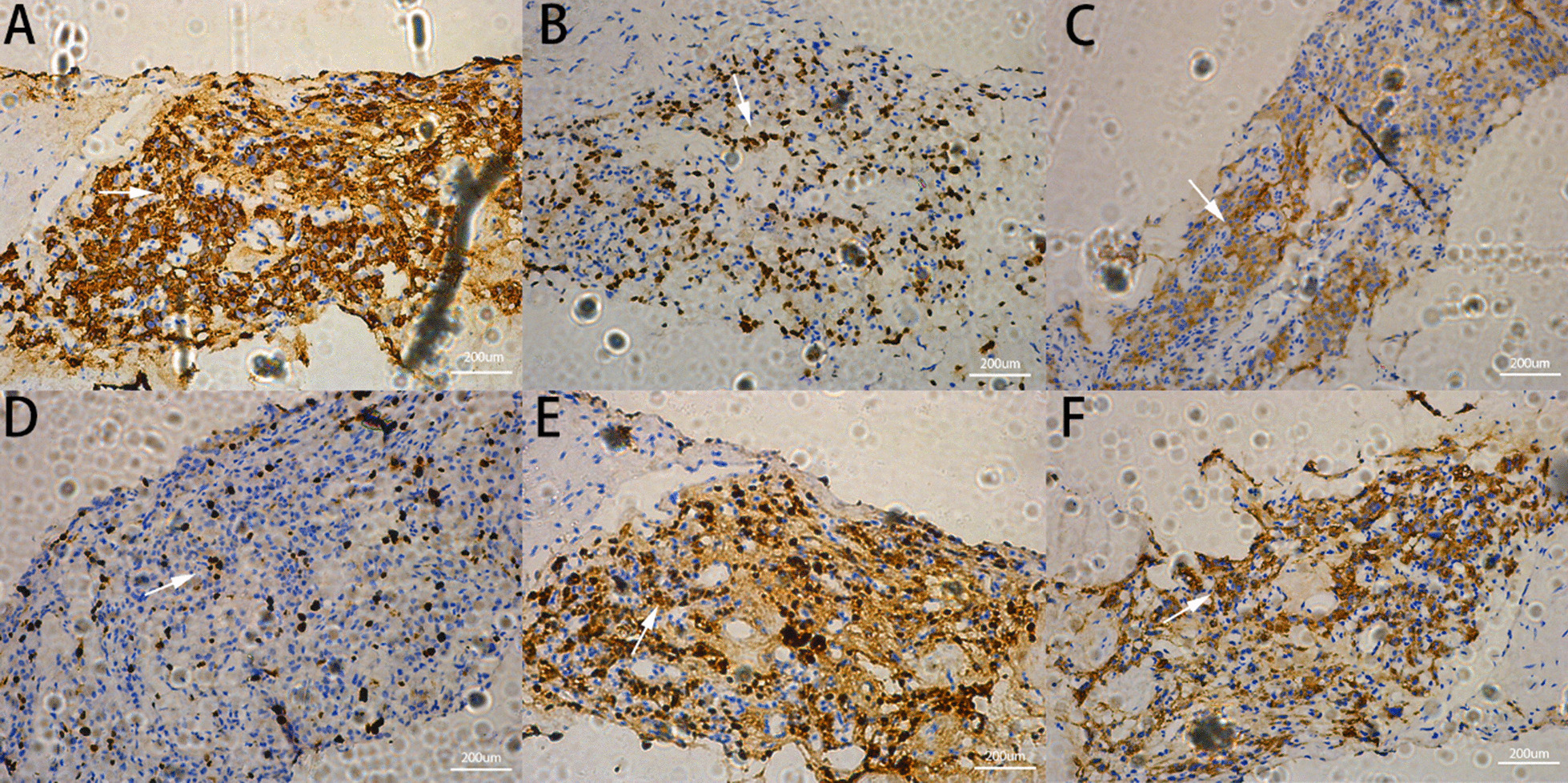
Positive immunohistochemical indicators. **A** CGA; **B** GATA-3; **C** inhibi-a; **D** Ki-67; **E** S-100; **F** SYN (magnification ×200). Type of equipment: microscope:LEICA DM 2000 LED; cameras: LEICA MC 170 HD. Acquisition software: LASV4.10

After colon cleansing, the patient underwent transrectal ultrasound-guided pelvic mass puncture. Pathological examination of the puncture specimen showed small nest-like cell masses in the fibrous connective tissue, with moderate volume, abundant pink staining or dichromatic cytoplasm, slightly enlarged nucleus and rare nuclear mitoses (Fig. 3). Immunohistochemical results showed CgA(+), SYN(+), S-100(+), GATA-3 (+), Ki-67 (approximately 10% +), and Inhibin-A (+). SMA, CD117, DOG, HMB45, TTF-1, CAM5.2, MELANA, AE1/AE3, WT1, D2-40, P63, and Calretinin were all negative. The findings were consistent with extra-adrenal paraganglioma (Fig. 4).

After treatment with oxygen inhalation, anti-infection, diuresis, vasodilation, blood pressure control, and blood glucose control, pleural effusion was absorbed and the symptoms of heart failure showed significant improvement. He was advised chemotherapy using cyclophosphamide, vincristine, and dacarbazine. However, the patient refused chemotherapy and died six months after leaving the hospital.

## Discussion

Functional paragangliomas are easily discovered because of the symptoms caused by excessive secretion of catecholamines and their metabolites, such as paroxysmal or persistent hypertension, hyperhidrosis, palpitations, and headache. Non-functional paraganglioma are typically detected when they cause symptoms of local compression. For example, patients with retroperitoneal or abdominal aortic paraganglioma often develop abdominal pain, abdominal discomfort, and back pain. Pelvic paragangliomas often cause urinary frequency and painless hematuria. Gastrointestinal paragangliomas may cause nausea, vomiting, constipation, and intestinal obstruction. In some patients, paragangliomas may be incidentally discovered during routine health examination. In this case, the patient developed symptoms of heart failure, and paraganglioma was discovered on ultrasound examination.

The diagnosis of extra-adrenal paraganglioma includes qualitative diagnosis and locational diagnosis. Determination of catecholamine levels (norepinephrine, epinephrine, dopamine) and their intermediate metabolites (metanephrine, methoxytryramine) and terminal metabolites (e.g. vanilmandelic acid) is an important basis for the qualitative diagnosis of paraganglioma [5, 6]. The sensitivity and specificity of measurement of catecholamines and their intermediate and terminal metabolites in plasma or urine for the qualitative diagnosis of paraganglioma are different. Among them, metanephrine and methoxytryramine have higher specificity and sensitivity [5, 7]. The levels of epinephrine and norepinephrine in this disease could be normal. Vanilmandelic acid is the final metabolite of epinephrine and norepinephrine, and its elevation is also significant for the diagnosis of disease [3, 5]. In this case, 24-h urinary vanilmandelic acid was elevated and adrenaline was normal, which is consistent with the characteristics of the disease. Pathological examination of non-functional tumors is necessary to prevent misdiagnosis.

Ultrasound, computed tomography, magnetic resonance imaging, ^123^I-MIBG and PET-CT are invaluable in locational diagnosis of paraganglionoma. In particular, ^123^I-MIBG has the highest specificity (95–100%) because it is an epinephrine analogue and can be absorbed by pheochromocyte catecholamine vesicles [8]. In this case, the lesion was detected on pelvic ultrasound. Subsequently, pelvic enhanced CT and ultrasound-guided transrectal pelvic mass puncture was performed to obtain the final pathological diagnosis.

In a case of rectal paraganglioma reported by Yu et al. [1], the tumor was composed of nested pleomorphic cells surrounded by bifurcated and fine blood vessels, forming characteristic Zellballen. Pleomorphic cells had abundant eosinophilic and amphiphilic granular cytoplasm, round or ovoid nuclei, and prominent nucleoli. There was tumor necrosis and vascular infiltration. On immunohistochemistry, the pleomorphic cells showed strong expression of pheochromogranin, synaptophysin, CD56, NSE, and vimentin [1]. The pathological results of this case were consistent with the pathological characteristics of most paragangliomas: nest-like distribution of cell masses, abundant pink or dichromatic cytoplasm, slightly enlarged nuclei, and rare mitoses. Studies have shown that most paragangliomas are S100 positive, synaptophysin (+), pheochromogranin(+), vimentin(+), and AE1 + AE3 negative. The immunohistochemical characteristics of this case were CgA(+), SyN(+), S-100(+), Cata-3(+), Ki-67(approximately 10% +), and Inhibin-A (+). SMA, CD117, DOG, HMB45, TTF-1, CaM5.2, Melana, AE1/AE3, WT1, D2-40, p63, and calretinin were all negative, which are also in accordance with the typical immunohistochemical results.

In 2017, the WHO proposed the term “metastatic paraganglioma” instead of “malignant paraganglioma”. The 2014 Endocrine Society clinical practice guidelines for pheochromocytoma and paraganglioma suggest that 10–17% of pheochromocytomas and paragangliomas are malignant, of which the proportion of paragangliomas is higher [5]. Malignant extra-adrenal paraganglioma is rare and reported to be less than 3%, of which nearly 75% are sporadic and 25% are hereditary [9]. Malignant tumors are mostly metastases or spread to organs without chromaffin tissue [10].

Paragangliomas are also categorized as benign and malignant. The distinction between benign and malignant is different from that in other tumors, and it does not depend on pathological diagnosis. At present, the diagnosis of malignant paraganglioma mainly depends on the presence or absence of intravascular tumor thrombus, local invasion, or lymph node metastasis. A study showed that histopathological findings of abundant intratumoral necrosis, vascular invasion, cystic invasion, and strong mitotic activity suggest the possibility of malignancy [11]. Some studies have also shown that the criteria for malignancy are tumor metastasis and tumor proliferation in non-chromaffin tissues. Factors associated with malignancy were large tumor size (> 5 cm), obvious nuclear pleomorphism, increased mitotic activity, tumor necrosis, lack of supporting cells, vascular invasion, diffuse surrounding structures and tissues, and high Ki67 index [3]. The grade of pheochromocytoma and paraganglioma, known as the GAPP score, is based on tumor pathology, cellular properties, acne-like necrosis, vascular or cystic infiltration, Ki67 index, and type of catecholamine secretion, with a maximum score of 10. A score of 0–2 indicates highly differentiated, 3–6 indicates moderately differentiated, and 7–10 indicates poorly differentiated. The 5-year survival rate was 100% for highly differentiated patients, 66.8% for moderately differentiated patients, and 22.4% for poorly differentiated patients [1]. Studies have shown that GAPP score is associated with survival or metastatic rates of paraganglioma [12].

This case was malignant paraganglioma, which was considered metastatic based on multiple retroperitoneal enlarged nodules (approximately 59 × 34 mm in diameter) detected on abdominal and pelvis enhanced CT. Bone ECT showed multiple spots, slices and strips of nuclide enhancement in the skull, vertebra, bilateral ribs, bilateral scapula, left upper humerus, pelvic bone, and right upper femur. These findings were suggestive of bone metastases.

Surgery is still the main treatment modality. Hypertension should be adequately controlled before surgery to prevent intraoperative hypertensive crisis or arrhythmia. Hemalatha reported a patient with retroperitoneal paraganglioma who died during the surgery because of hypertensive crisis [13]. Radiotherapy and chemotherapy can be used for patients with metastases. I-MIBG is ingested by pheochromocyte vesicles, leading to emission of β rays, which can act on pheochromocytes to achieve therapeutic effect. This patient disapproved of chemotherapy for personal reasons, and died within six months of diagnosis.

The possibility of neuroendocrine-related tumors, for example paragangliomas, should be considered in young patients with heart failure, especially those with concomitant hypertension and diabetes. Better characterization of the disease can facilitate early diagnosis and help improve treatment outcomes.

## Data Availability

All data generated or analysed during this study are included in this published article.

## Abbreviations

WHO: World Health Organization
CT: Computed tomography
PET-CT: Position emission tomography-computed tomography
VMA: Vanillylmandelic acid
ECT: Emission computed tomography
BNP: B-type natriuretic peptide
GAPP: Grading system for adrenal pheochromocytoma and paraganglioma
^123^I-MIBG: ^123^I-Metaiodobenzylguanidine
CgA: ChromograninA
SYN: Synaptophysin
S-100: S100 calcium binding protein
GATA-3: Transcription factor encoded in humans by the GATA3 gene
SMA: Smooth muscle actin
CD117: Proto-oncogene tyrosine-protein kinase Kit
WT1: Wilms tumor protein
TTF-1: Thyroid transcription factor-1
NSE: Neuron specific enolase
AE1/AE3: Monoclonal mouse anti cytokeratin
CD 56: Archetypal phenotypic marker of natural killer
Ki-67: The proliferation marker
